# Orbital Compartment Syndrome as a Complication of Blepharoplasty: A Case Report

**DOI:** 10.5811/cpcem.42014

**Published:** 2025-09-10

**Authors:** Lev Libet, Jairo Garcia, Lawrence Liu

**Affiliations:** Kern Medical, Department of Emergency Medicine, Bakersfield, California

**Keywords:** lateral canthotomy, cantholysis, blepharoplasty, acute vision loss

## Abstract

**Introduction:**

Acute vision loss constitutes a true medical emergency, as a delay in diagnosis and treatment may lead to permanent visual impairment. Orbital compartment syndrome is most commonly associated with blunt trauma causing a retro-orbital hematoma and resulting compromise of the optic nerve. Orbital compartment syndrome, however, can occur in other scenarios including status post blepharoplasty.

**Case Report:**

This is a case of a 67-year-old male who presented less than 24 hours after a bilateral upper blepharoplasty due to decreased visual acuity of his right eye. A lateral canthotomy was performed despite the absence of elevated intraocular pressures on tonometry. He regained visual acuity in his right eye shortly after the cantholysis.

**Conclusion:**

It is vital to consider the range of entities that can cause orbital compartment syndrome, including blepharoplasty. Recognition and emergent intervention improved the visual acuity in this case.

## INTRODUCTION

Visual complaints comprise approximately 0.7% of emergency department (ED) visits. Of these, 41.2% are categorized as emergent.^1^ The ocular nerve is susceptible to injury when pressure builds in the retro-orbital space. This classically occurs in the setting of blunt trauma that causes a retro-orbital hematoma. As the hematoma expands so does the pressure in the orbital compartment. The optic nerve is compressed leading to compromised vascular flow, which results in visual impairment. In orbital compartment syndrome, visual changes are often noted when the ocular pressure is greater than 40 millimeters of mercury (mmHg).^2^

Blepharoplasty is a common cosmetic outpatient procedure performed by ophthalmologists or plastic surgeons; on rare occasions it can result in orbital compartment syndrome. We present a case of acute vision loss status post blepharoplasty that improved after cantholysis.

## CASE REPORT

A 67-year-old male presented to the ED with a complaint of bleeding and rapid swelling of his right lower eyelid. The patient had undergone bilateral upper blepharoplasty at an outpatient surgery center one day prior to presentation. After the procedure he felt well, was without complaints, and was released to recover at home. One hour prior to arrival to the ED, the patient noted significant bleeding from the right lower eyelid. He reported diplopia initially, which progressed to blurred vision on the right. By the time of his arrival to the ED, he reported seeing only shadows and movement and had a constant pressure sensation to his right eye. He was not taking anticoagulants, nor did he experience any preceding trauma.

**Image 1:**
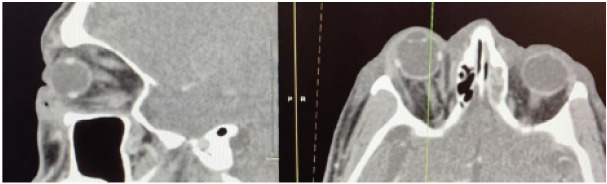
Computed tomography of the orbits with the axial view on the right and sagittal view on the left. The vertical yellow line delineates the sagittal plane shown on the left. Proptosis of the right eye can be seen on the axial view without evidence of retrobulbar hematoma or active hemorrhage.

Examination of the right eye was notable for right periorbital edema and ecchymosis predominantly of the lower lid, chemosis, and mild proptosis of the globe. Eyelid sutures were clean, intact, and without active hemorrhage. The right pupil was fixed and dilated at 5 mm, and ophthalmoplegia was noted to the right eye in all directions. Intraocular pressures (IOP) were measured using an iCare™ IC100 tonometer (iCare USA, Inc, Raleigh, NC): oculus dexter (OD) 14 mmHg (reference range: 0–20 mmHg); oculus sinister (OS) 10 mmHg. Light perception was present only at the nasal upper quadrant. Due to the mismatch of examination and measured IOP, we ordered a computed tomography (CT) with intravenous contrast of the orbits. The CT demonstrated right eye proptosis with extraconal periorbital and infraorbital soft tissue swelling without demonstration of active hemorrhage or hematoma (Image 1).

Due to clinical findings of proptosis, vision loss, and ophthalmoplegia, the emergency medicine resident performed a lateral canthotomy followed by cantholysis of both the inferior and superior crus of the right eye. After cantholysis, the patient endorsed immediate improvement of the sensation of pressure and gradual return of visual acuity. The right pupil normalized to 3 mm in diameter, was reactive to light, and ophthalmoplegia resolved. Post cantholysis his visual acuity was OD 20/70, and OS 20/40. Ophthalmology was consulted and recommended that the patient be discharged back to his ophthalmologist for further evaluation.

*CPC-EM Capsule*

What do we already know about this clinical entity?

*Orbital compartment syndrome is an expected complication of a retroorbital hemorrhage resulting from facial trauma*.

What makes this presentation of disease reportable?

*Orbital compartment syndrome as a result of a cosmetic surgery such as blepharoplasty is rarely seen in the emergency department*.

What is the major learning point?

*The clinical presentation in its totality is more important than a single data point, in this case the ocular pressure measurement*.

How might this improve emergency medicine practice?

*This case will hopefully increase awareness of the possibility of orbital compartment syndrome as a complication of blepharoplasty*.[Fig f2-cpcem-9-389]

## DISCUSSION

Blepharopasty is a commonly performed cosmetic procedure that entails removal of excess skin from either the upper or lower eyelid. The procedure is performed in the outpatient setting and is relatively safe. Some of the potential complications include lagophthalmos, which is failure of the upper lid to reach the lower lid, ectropion, or turning outward of the lower lid, and dry eyes. The complications that require emergent intervention are acute angle closure glaucoma and orbital compartment syndrome.^3^,^4^ Orbital compartment syndrome due to blepharoplasty procedure was first described in 1981.^5^ Our patient’s presentation was initially concerning for a retrobulbar hematoma secondary to postoperative bleeding causing acute orbital compartment syndrome. The presence of pain, a fixed mydriatic pupil, and vision loss made angle closure glaucoma a consideration but less likely, given the findings of chemosis and proptosis. The normal IOP did not support either etiology—orbital compartment syndrome or angle closure glaucoma.

While retrobulbar hematoma is a rare complication, it accounts for 51% of cases of acute vision loss after blepharoplasty.^3^ Orbital compartment syndrome will usually present within 12 hours of the procedure but may occur up to nine days after blepharoplasty.^6^ A deep orbital hemorrhage is the likely etiology in this case. This occurs as a complication in 0.05% of blepharoplasties and causes vision loss in 0.01%.^7^ When orbital compartment syndrome is diagnosed, patients who receive intervention within two hours have an increased likelihood of improved visual acuity compared with those delayed beyond that time frame.^8^ While the patient in the case presented had immediate resolution of visual symptoms, it is worth noting that improvement in visual acuity may take up to several weeks.^8^ The presentation aligns with orbital compartment syndrome despite the absence of a clear retrobulbar hematoma and the presence of a normal ocular pressure. It is possible that there was a calibration error of our tonometer, but otherwise it is unclear why the measured IOP was normal. This case highlights the importance of relying on the complete clinical picture and not on any single test.

## CONCLUSION

The complication of orbital compartment syndrome can occur rarely in patients who have undergone recent blepharoplasty. The diagnosis requires emergent intervention as the duration of optic nerve compression is correlated with the outcome of visual acuity. In this case, the intraocular pressure was not elevated; however, the clinical picture dictated the need for emergent intervention. Cantholysis was performed by the emergency medicine service, and our patient had rapid improvement in his visual acuity.

## Figures and Tables

**Image 2 f2-cpcem-9-389:**
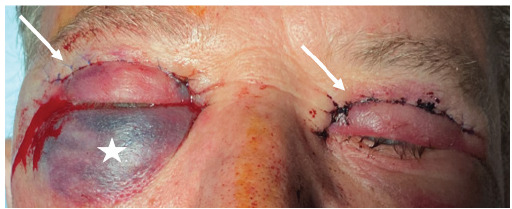
Image of eye lids after cantholysis on the right side, depicting edema and ecchymosis to the lower lid on the right (white star) and sutures to the bilateral upper palpebrae (white arrows).
